# A Validated Stability-Indicating Liquid Chromatographic Method for Determination of Degradation Impurities and Diastereomers in Voriconazole Tablets

**DOI:** 10.3797/scipharm.1204-24

**Published:** 2012-06-18

**Authors:** Kabeer A. Shaikh, Ashish T. Patil

**Affiliations:** Dr. Babasaheb Ambedkar Marathwada University, Department of Chemistry, Sir Sayeed College, Aurangabad, 431001, M.S., India.

**Keywords:** Development, Validation, HPLC, Voriconazole, Forced Degradation study

## Abstract

A reversed-phase gradient liquid chromatographic method has been developed for the quantitative determination of Voriconazole, along with its degradation and diastereomeric impurities in tablet dosage form. Chromatographic separation has been achieved on an Inertsil ODS 3V, 150 × 4.6 mm, 5 μm column. The mobile phase consisting of solvent A 0.05 molar (M) potassium dihydrogen phosphate (pH 2.5 buffer) and solvent B (mixture of acetonitrile and methanol in the ratio 90:10 (*v/v*)), was delivered at a flow rate of 1.2 mL min^−1^ with the detection wavelength at 256 nm. Resolution of Voriconazole and all five potential impurities was achieved at greater than 2.0 for all pairs of compounds. The drug was subjected to stress conditions such as oxidative, acid and base hydrolysis, and thermal and photolytic degradation. Voriconazole was found to degrade significantly under base hydrolysis stress conditions compared to acid hydrolysis stress conditions. The degradation products were well-resolved from the main peak and its impurities, thus proving the stability-indicating power of the method. The stressed samples were assayed against a reference standard and the mass balance was found to be close to 99.0%. The developed method was validated as per ICH guidelines with respect to specificity, linearity, limit of detection, limit of quantification, accuracy, precision, and robustness.

## Introduction

**Fig. 1.**
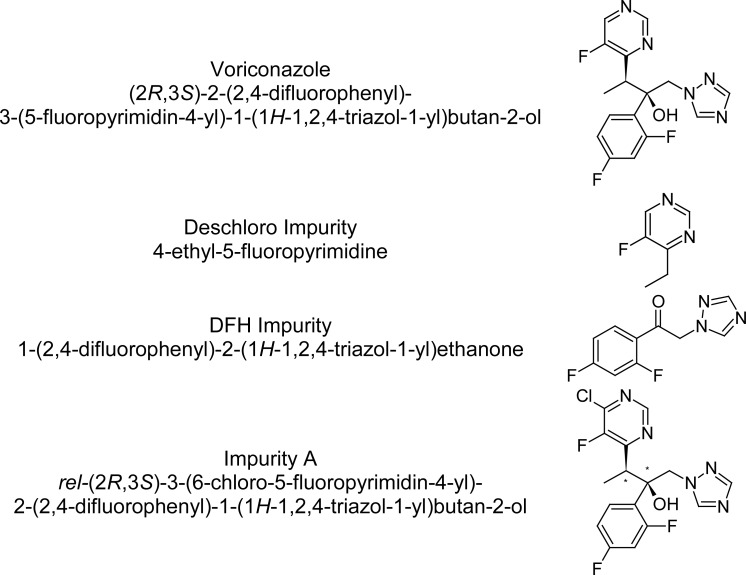
Chemical structure of Voriconazole and its impurities.

Voriconazole belongs to a class of antifungal medicines used to treat serious and invasive fungal infections, which are generally seen in immunocompromised patients. Its chemical designation is (2*R*,3*S*)-2-(2,4-difluorophenyl)-3-(5-fluoropyrimidin-4-yl)-1-(1*H*-1,2,4-triazol-1-yl)butan-2-ol (Figure 1). The possible related compounds, degradents, and process-related impurities of Voriconazole are shown in Figure 1. Voriconazole is commercially available in two strengths, 50 mg and 200 mg, with the brand name VFend (Pfizer, Inc.). So far, some liquid chromatographic procedures have been described for the determination of Voriconazole in tablet dosage form [1–4, 9, 10]. The HPLC method is used for the quantification of Voriconazole in plasma [5]. The determination of Voriconazole in pharmaceutical formulations is possible using an experimental design [6]. Also informative is the measurement of Voriconazole in serum and plasma [8]. To the best of our knowledge, all of the above analytical methods are used only for the quantification of Voriconazole, not for the quantification of known related compounds and degradation impurities of Voriconazole in Tablet dosage form. Voriconazole degrades significantly under base hydrolysis stress conditions as compared with acid hydrolysis [2, 4, 7]. The major degradation impurities observed are deschloro and DFH. One HPLC method is available for the determination of Voriconazole and its related substances [7], but this method does not address the chromatographic separation of the diastereomers belonging to Voriconazole along with degradation impurities. Diastereomers are compounds with the same chemical structure as the desired molecule, but differ in structural arrangement. Chemical sameness creates difficulty in separating diastereomers, but this proposed method is capable of separating diastereomers in the Voriconazole tablet dosage form.

A literature survey reveals that the Voriconazole Tablet is not official in any pharmacopeia. None of the currently available analytical methods are capable of separating all of the degradation impurities or process-related impurities in the Voriconazole tablet dosage form. The base degradation of Voriconazole may seriously affect the quality of products, and is usually associated with a reduction of the pharmacological activity and/or the occurrence of side effects. The stress conditions are useful for establishing degradation pathways, and for developing and validating suitable procedures. This present work describes analytical parameters aimed to achieve an alternative for the quantification of Voriconazole and its degradation products along with diastereomers in tablets dosage forms, in accordance with ICH recommendations [11].

## Experimental

### Chemicals and reagents

All standards and tablets were purchased from the Indian market (Vorizol tablets by Natco Pharmaceuticals Ltd.). The HPLC grade acetonitrile, methanol, and analytical grade potassium dihydrogen phosphate, and *ortho*-phosphoric acid were purchased from Merck. The water used was obtained by using the Millipore MilliQ Plus water purification system.

### Equipment

The chromatographic system used was an Agilent-1100 series comprised of a degasser, quaternary pump, auto injector, column compartment, PDA detector, and the photostability studies were carried out in a photostability chamber (Sanyo, Leicestershire, UK). Thermal stability studies were performed in a dry air oven (Cintex, Mumbai, India).

### Chromatographic Conditions

The chromatographic column used was an Inertsil ODS 3V 150x4.6mm, 5μm. The separation was achieved by a gradient method. The 0.05 molar (M) potassium dihydrogen phosphate (pH 2.5) buffer and the mobile phase B contained a mixture of acetonitrile and methanol in the ratio 90: 10 (*v/v*); respectively. The flow rate was 1.2 mL min^−1^. The HPLC gradient program was set as time (min) / % solution B: 0/20, 20/40, 38/60, 40/20, and 45/20. The column temperature was maintained at 35 °C and the detection was monitored at a wavelength of 256 nm. The injection volume was 20 μL. A mixture of water and acetonitrile in the proportion of 50:50 (*v/v*); respectively, was used as a solvent or diluent.

### Preparation of standard Solutions

A stock solution of Voriconazole (300 μg mL^−1^) was prepared by dissolving an appropriate amount in the solvent mixture. Standard solutions containing 3 μg mL^−1^ were prepared from this stock solution.

### Preparation of Sample Solution

The tablet powder equivalent to 50 mg drug was dissolved in solvent with rotary shaking for 10 min and then sonicated for 10 min to give a solution containing 1000 μg mL^−1^. This solution was filtered through a 0.45 μm pore size Nylon 66 membrane filter.

## Results and Discussion

### Method Development and Optimization

The important criteria for the development of the successful RP-HPLC method for the determination of Voriconazole-related substances in tablet dosage form, was the method which should be able to determine all impurities of the drug in single run with a good amount of resolution. The method should be accurate, reproducible, robust, stability-indicating, free from interference (blank/placebo/ other unknown degradation product), and straightforward enough for routine use in quality control laboratories. The main objective of the chromatographic method development was to separate Voriconazole impurities from the main peak with a good amount of resolution. The initial method development started with an Isocratic mobile phase. Different combination of buffers: acetonitrile in the range of 90:10 to 10: 90 v/v were used, and it was observed that the deschloro impurity and DFH impurity are mostly polar in nature, whereas impurity A is nonpolar in nature. Then an increase in the buffer concentration of more than 50% in the mobile phase lead to more retention of impurity A on the column, which lead to an increased run time of more than 60 minutes. Also, the peak shape of impurity A is not proper. By a decrease in buffer concentration to less than 50% in the mobile phase, retention of impurity A was reduced, but the resolution between the deschloro and DFH impurities also decreased, where both peaks eluted to nearly a void volume. As a result, the gradient mobile phases were switched, where potassium dihydrogen phosphate (pH 2.5) buffer was used as mobile phase A, and mobile phase B used acetonitrile and methanol in the ratio 90:10 v/v. Different gradient programs have been attempted to improve the run time to be less than 60 minutes with good retention of the deschloro and DFH impurities on the column. The peroxide stressed sample and impurity-spiked sample were injected in the column to check for good resolution between the known and unknown impurities, and Voriconazole.

During the optimization of the method, the gradient program has been finalized as time (min) / % solution B: 0/20, 20/40, 38/60, 40/20, and 45/20. The column temperature was maintained at 35 ºC and the detection was monitored at a wavelength of 256 nm. The injection volume was 20 μL. The relative response factor for all five impurities was determined with respect to Voriconazole (Table 1).

### Analytical parameters and validation

The optimized chromatographic conditions were validated by evaluating specificity, linearity, precision, accuracy, limit of detection (LOD), limit of quantification (LOQ), robustness, and system suitability in accordance with ICH guidelines Q2 (R1) [12].

#### Specificity

The specificity of a method is its suitability for the analysis of a substance in the presence of potential impurities. Stress testing of a drug substance can help to identify likely degradation products, which can help to establish degradation pathways and the intrinsic stability of the molecule. It can also be used to validate the stability-indicating power of the analytical procedures used. The specificity of the LC method for Voriconazole has been determined in the presence of five impurities.

#### Forced Degradation Study of Drug Product

The stress degradation study was performed on the drug product, which includes acid hydrolysis (5ml of 0.1 M HCl at 60°C for 2hr), base hydrolysis (5ml of 0.1 M NaOH at 60°C for 30min), oxidation (2ml of 1% H_2_O_2_ on bench top for 30min), thermal (60°C for 24hrs), humidity (40°C, 75% RH for 7 days), and photolytic degradation (drug product exposed to UV and visible light so it has to complete 1.2 million lux hours and 200 watt h/m^2^). The stress study was performed as per the International Conference on Harmonization (ICH) recommendation. Peak purity was checked for the Voriconazole peak by using a PDA detector in stress samples. An assay of stressed samples was performed for comparison to the reference standard, and the mass balances (% assay + % impurities + % degradation products) were then calculated. No peaks were found at the retention time of Voriconazole and its five impurities in blank and placebo chromatograms, which proves there was no interference from the blank and placebo. Slight degradation was observed when the drug product was subjected to acid hydrolysis and peroxide stress conditions, and was stable in photolytic and humidity stress conditions. Voriconazole was sensitive in basic and heat conditions, and it significantly degraded into the deschloro and DFH impurities (Figures 3–5). Peak purity test results from the PDA detector confirmed that the Voriconazole peak obtained from all of the stress samples analyzed, was homogeneous and pure. Peak purity results from the PDA detector for the peaks produced by the degradation of Voriconazole, confirmed that all these peaks were homogeneous and pure for all the stress samples analyzed (Table 2). The mass balance for the stressed samples was close to 99% (Table 2). The assay of Voriconazole was unaffected by the presence of the impurities/degradation products, confirming the stability-indicating power of the method.

#### Limits of Detection and Quantification

The LOD and LOQ for the five impurities and Voriconazole were estimated as the amounts for which the signal-to-noise ratios were 3:1 and 10:1, respectively, by injecting a series of dilute solutions of known concentration. Precision was also determined at the LOQ level by analysis of the six individual preparations of the five impurities, and by calculating the RSD (%) of the peak area for each impurity.

#### Linearity

Solutions for testing the linearity of the related substances were prepared by diluting the impurity stock solution to five different concentrations from the LOQ to 200% of the permitted maximum level of the impurity (i.e. the LOQ to 0.6% for Voriconazole and all impurities for an analyte concentration of 1000 μg mL^−1^). The correlation coefficients, slopes, and *y*-intercepts of the calibration plots are reported. Calibration plots for the five related substances were linear over the ranges tested. The correlation coefficients were >0.999 for all of the components (Table 3). These results show that there was an excellent correlation between the peak area and concentration for the five impurities.

#### Precision

The precision of the method was verified by repeatability and intermediate precision. Repeatability was checked by injecting five individual preparations of the real sample of Voriconazole spiked with 0.30% of its five impurities. The intermediate precision of the method was also evaluated using a different analyst and a different instrument, and performing the analysis on different day. %RSD of area for each impurity was calculated for both precision as well as intermediate precision, and was found to be within 2%. These results confirmed the precision and ruggedness of the method (Table 3).

#### Accuracy

**Fig. 2.**
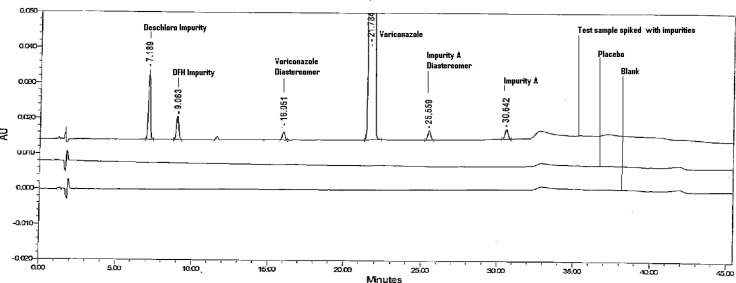
A Typical HPLC Chromatogram Placebo, Blank, and Sample solution spiked with impurities.

**Fig. 3.**
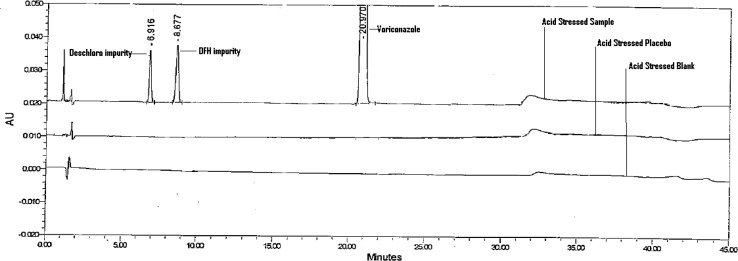
A Typical HPLC Chromatogram of Acid Stressed Blank,Placebo, and Sample.

**Fig. 4.**
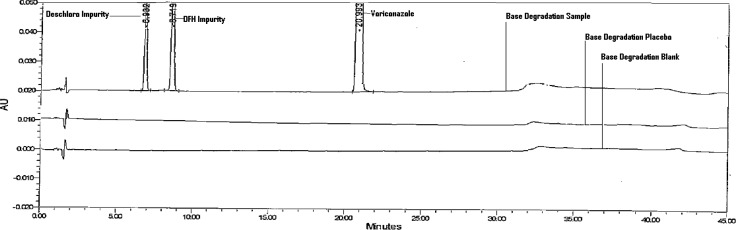
A Typical HPLC Chromatogram of Base Stressed Blank, Placebo, and Sample.

**Fig. 5.**
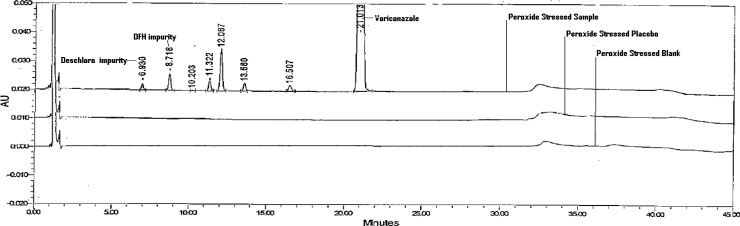
A Typical HPLC Chromatogram of Peroxide Stressed Blank, Placebo, and Sample.

For the impurities, recovery was determined in triplicate for LOQ, 0.15, 0.30, and 0.45% of the analyte concentration (1000 μg mL^−1^) for Voriconazole, and then recovery of the impurities was also calculated (Table 4). An HPLC chromatogram obtained from a sample of Voriconazole spiked with all five impurities at the 0.30% level is shown in Figure 2.

#### Robustness

To determine the robustness of the method, the experimental conditions were deliberately changed and the resolution of Voriconazole and the five impurities was evaluated. In order to study the effect of flow rate on resolution, it was changed to 1.0 and 1.4 mL min^−1^. The effect of pH was studied at pH 2.3 and 2.7. The effect of column temperature was studied at 30 and 40 °C. In all of these experiments the mobile phase components were not changed. The effect of the percent organic strength on resolution was studied by varying acetonitrile by −10 to +10%, while other mobile phase components were held constant. In all of the deliberately varied chromatographic conditions, the selectivity as well as the performance of the method were unchanged, which proves the robustness of the method.

#### Stability in Solution and in the Mobile Phase

No significant changes in the amounts of the five impurities were observed during the solution stability and mobile phase stability experiments when performed using the related substances method. The results from the solution stability and mobile phase stability experiments confirmed that standard solutions and samples were stable for up to 24 h during the determination of related substances. The mobile phase was stable up to 48h.

## Conclusion

The gradient RP-HPLC method developed for quantitative analysis of Voriconazole and related impurities in tablet dosage form is precise, accurate, linear, robust, rugged, and specific. Satisfactory results were obtained from validation of the method. The method is stability-indicating and can be used for routine analysis of production samples, and to check the stability of Voriconazole in tablet dosage form.

## Figures and Tables

**Tab. 1.** Chromatographic performance data.

**Compound**	**RT (min)**	**RRT^a^ (n = 3)^d^**	**Resolution^c^ (n = 3)^d^**	**Tailing factor (n = 3)^d^**	**RRF^b^**
Deschloro impurity	7.189	0.33	–	1.01	1.3
DFH/TAP	9.063	0.42	8.7	1.08	1.7
Voriconazole Diasterio.	16.051	0.74	22.3	1.09	0.8
Voriconazole	21.784	1.00	16.9	1.02	1.0
Impurity A Diasterio.	25.559	1.17	10.5	1.14	0.9
Impurity A	30.642	1.41	13.9	1.16	1.1

a Relative retention times (RRT) were calculated against the retention time of Voriconazole;

b Relative response factor were calculated against the response factor of Voriconazole;

c Resolutions were calculated between two adjacent peaks;

d Mean ± SD.

**Tab. 2.** Stress testing (forced degradation) data of Voriconazole.

**Stress Condition**	**%Net Degradation**	**Purity Angle**	**Purity threshold**	**Purity flag**	Mass Balance^*^
Acid Hydrolysis	1.6%	0.341	0.873	No	99.8
Base Hydrolysis	7.2%	0.567	0.912	No	99.5
Peroxide Oxidation	1.4%	0.456	0.767	No	99.1
Photolytic-sunlight	Stable	0.256	0.834	No	99.5
Heat Stress	5.0%	0.342	0.781	No	99.2
Humidity Stress	Stable	0.456	0.621	No	99.8

* Mass balance = % assay + % impurities + % degradation products.

**Tab. 3.** Regression and precision data.

**Parameters**	**Voriconazole**	**Deschloro**	**DFH**

LOQ μg mL^−1^	0.25	0.22	0.15
LOD μg mL^−1^	0.09	0.06	0.05
Regression equation (y)			
Slope (b)	21285.2	28308.9	36980.4
Intercept (a)	196.3	271.0	373.4
Correlation coefficient	0.9998	1.0000	1.0000
Precision (% RSD)^a^	1.5	1.2	1.6
Intermediate precision (% RSD)^a^	1.6	1.5	1.3
Precision @ LOQ (% RSD)^a^	1.2	1.3	1.7

**Parameters**	**Voriconazole Diastereomer**	**Imp.A Diastereomer**	**Imp. A**

LOQ μg mL^−1^	0.30	0.28	0.26
LOD μg mL^−1^	0.08	0.07	0.07
Regression equation (y)			
Slope (b)	18197.2	19278.9	24032.3
Intercept (a)	421.4	77.3	270.7
Correlation coefficient	0.9996	0.9999	0.9997
Precision (% RSD)^a^	1.8	1.4	1.7
Intermediate precision (% RSD)^a^	1.6	1.5	1.1
Precision @ LOQ (% RSD)^a^	1.2	1.3	1.2

Linearity range is LOQ – 200% with respect to 0.3% specification level;

a Six determinations.

**Tab. 4.** Evaluation of accuracy.

**Amount Spiked^a^**	**% Recovery^b^ Voriconazole**	**Deschloro**	**DFH**	**Voriconazole Diaster.**	**Impurity A Diaster.**	**Impurity A**
LOQ	99.7 ± 0.11	98.9 ± 0.12	99.5 ± 0.08	98.4 ± 0.23	98.4 ± 0.21	99.0 ± 0.54
50%	98.9 ± 0.31	98.6 ± 0.31	98.2 ± 0.43	99.7 ± 0.44	99.1 ± 0.37	98.9 ± 0.25
100%	99.9 ± 0.10	99.1 ± 0.16	99.4 ± 0.01	99.0 ± 0.56	99.7 ± 0.65	98.4 ± 0.16
150%	99.2 ± 0.21	99.4 ± 0.56	99.5 ± 0.31	98.6 ± 0.61	99.2 ± 0.41	98.8 ± 0.14

a Amount of five impurities spiked with respect to 0.30% specification level of Voriconazole;

b Mean ± % RSD for three determinations.
